# The biological functions and metabolic pathways of valine in swine

**DOI:** 10.1186/s40104-023-00927-z

**Published:** 2023-10-07

**Authors:** Chuni Wang, Yao Peng, Yiru Zhang, Juan Xu, Sheng Jiang, Leli Wang, Yulong Yin

**Affiliations:** 1 Institute of Subtropical Agriculture, Chinese Academy of Sciences, Changsha, China; 2 Hunan Provincial Key Laboratory of Animal Intestinal Function and Regulation, College of Life Sciences, Hunan Normal University, Changsha, China; 3 Tianjin Institute of Industrial Biotechnology, Chinese Academy of Sciences, Tianjin, China

**Keywords:** Additional dosage, Biological function, Metabolic pathway, Pig, Valine

## Abstract

Valine is an essential amino acid and a type of branched-chain amino acid. Due to the involvement of branched-chain amino acids in various metabolic pathways, there has been a surge of interests in valine nutrition and its role in animal physiology. In pigs, the interactions between valine and other branched-chain amino acids or aromatic amino acids are complex. In this review, we delve into the interaction mechanism, metabolic pathways, and biological functions of valine. Appropriate valine supplementation not only enhances growth and reproductive performances, but also modulates gut microbiota and immune functions. Based on past observations and interpretations, we provide recommended feed levels of valine for weaned piglets, growing pigs, gilts, lactating sows, barrows and entire males. The summarized valine nutrient requirements for pigs at different stages offer valuable insights for future research and practical applications in animal husbandry.

## Introduction

The basic function of amino acids is to synthesize proteins in bodies [1]. They can also play the non-proteinogenic functions as bioactive molecules in nutrition metabolism, stress response, and tissue development [1, 2]. Many amino acids have showed versatile biochemical properties and functions for swine due to variations in their side chains [3, 4]. Valine, leucine and isoleucine belong to branched-chain amino acids (BCAAs) because their functional R groups are all branched [5]. It has been reported that the addition of BCAAs in piglet diet has a positive effect on muscle mass and protein synthesis [6]. Valine cannot be de novo synthesized by animals, and it must to be obtained through protein degradation from diet, such as grains and fish meal [7–9]. Unlike *D*-valine, which forms bacterial cell walls, *L*-valine is more widely used to synthesize proteins in the body [10–12]. In the swine industry, *L*-valine is commonly used as a white crystalline or crystalline powder [13, 14].

Ammonia, biogenic amines, and indolic compounds belong to protein fermentation metabolites, which increase colon permeability and damage intestinal health [15]. For farm animals, low protein (LP) diet is a diet pattern in which the crude protein (CP) level is reduced by 2% to 4% without affecting the growth performance, and increases the appropriate dosages of limiting amino acids [16, 17]. The LP diet improves nitrogen utilization rate and limits environmental pollution caused by nitrogen excretion in intensive animal livestock production [18–20]. Sometimes, the reduction of dietary CP may lead to the increase in endogenous synthesis of non-essential amino acids for the nitrogen requirements [21]. Lysine, threonine, methionine, and tryptophan, as the four limiting amino acids, have been widely added in the LP diet to balance for an ideal protein ratio and meet the requirements for essential amino acids and total nitrogen in growing-finishing pigs [21]. In lactating sows, the second limiting amino acid varies with the change of tissue mobilization, while lysine is consistently regarded as the first limiting amino acid [22].

Similar to the above limiting amino acids, in the swine industry, supplementation with crystalline valine also maintains growth performance by supplying with a more balanced amino acid profile in the corn-soybean diet with low CP [14, 23]. As has been neglected for growing-finishing pigs in the past, valine is regarded as the fifth limiting amino acid [21]. When lactating pigs do not mobilize body tissue, valine acts as the second limiting amino acid [22]. In pig nutrition, the interactions have been observed between valine with isoleucine and leucine, as well as between valine with aromatic amino acids [14, 24]. According to its metabolic pathways, this review emphasized the roles of valine to regulate energy supply, structure of gut microbiota, immune functions, and reproductive performance in swine. The recommended nutrient requirements of valine at pig different growth stages were summarized in Table 1 [25–27]. The comprehensive analysis of the valine supplementation is still an evolving aspect of study in swine, and targeted application of valine to support animal demands should be valued in subsequent research.

**Table 1:** Some recommended standard content of SID valine/lysine in diets for swine

SID valine:lysine	Category and body weight	References
64%	Piglets	NRC 2012 [25]
70%	Piglets	British Society of Animal Science 2003 [26]
86.3%	Weaned piglets (5 to 10 kg)	Mavromichalis et al. [27]
63.3%	Growing pigs (5 to 7 kg)	NRC 2012 [25]
63.7%	Growing pigs (7 to 11 kg)	NRC 2012 [25]
67.4%	Growing pigs (10 to 20 kg)	Mavromichalis et al. [27]
63.4%	Growing pigs (11 to 25 kg)	NRC 2012 [25]
70.3%	Growing pigs (15 to 30 kg)	British Society of Animal Science 2003 [26]
65.3%	Growing pigs (25 to 50 kg)	NRC 2012 [25]
70.2%	Growing pigs (30 to 60 kg)	British Society of Animal Science 2003 [26]
64.7%	Growing pigs (50 to 75 kg)	NRC 2012 [25]
70.6%	Growing pigs (60 to 90 kg)	British Society of Animal Science 2003 [26]
65.8%	Growing pigs (75 to 100 kg)	NRC 2012 [25]
70.4%	Growing pigs (90 to 120 kg)	British Society of Animal Science 2003 [26]
67.2%	Growing pigs (100 to 135 kg)	NRC 2012 [25]
65.5%	Gilts (50 to 75 kg)	NRC 2012 [25]
66.2%	Gilts (75 to 100 kg)	NRC 2012 [25]
67.2%	Gilts (100 to 135 kg)	NRC 2012 [25]
73.5%	Sows and gilts	British Society of Animal Science 2003 [26]
76.6%	Lactating sows	British Society of Animal Science 2003 [26]
76.2%	Lactating gilts	British Society of Animal Science 2003 [26]
65.4%	Barrows (50 to 75 kg)	NRC 2012 [25]
66.7%	Barrows (75 to 100 kg)	NRC 2012 [25]
67.2%	Barrows (100 to 135 kg)	NRC 2012 [25]
65.9%	Entire males (50 to 75 kg)	NRC 2012 [25]
65.9%	Entire males (75 to 100 kg)	NRC 2012 [25]
65.8%	Entire males (100 to 135 kg)	NRC 2012 [25]

*NRC* National research council, *SID* Standardized ileal digestibility

### Metabolic pathways of valine

**Fig. 1:**
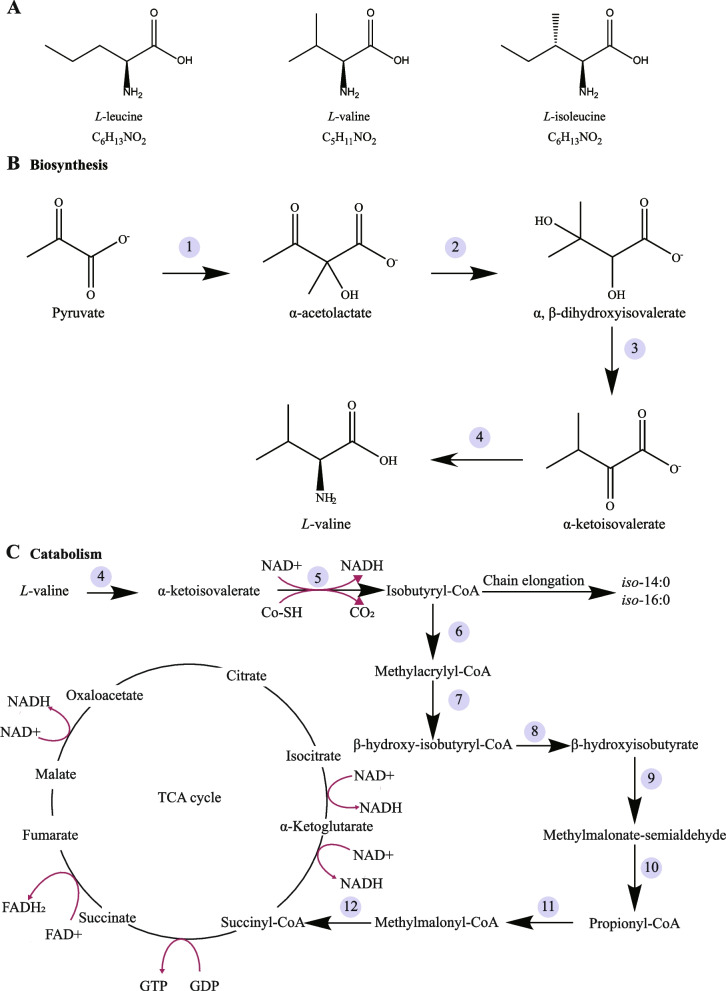
Structure and pathways. **A** Structure of BCAAs, including *L*-leucine, *L*-valine and *L*-isoleucine. **B** An overview of biosynthetic pathway of *L*-valine in bacteria, such as *Corynebacterium glutamicum* and *Escherichia coli*. **C** A schematic diagram for the complete metabolism of *L*-valine in animals. *L*-valine is catabolized to succinyl-CoA and subsequently enter the TCA cycle. The numbers correspond to the appropriate enzymes for main reactions. 1. Acetohydroxy acid synthase; 2. Acetohydroxy acid isomeroreductase; 3. Dihydroxy-acid dehydratase; 4. Branched chain amino acid transaminase; 5. Branched chain keto-acid dehydrogenase; 6. 2-Methylbutyryl-CoA dehydrogenase; 7. Enol-CoA dehydrogenase; 8. 3-Hydroxyisobutyryl-CoA deacylase; 9. 3-Hydroxyisobutyryl-CoA dehydrogenase; 10. Methylmalonic semialdehyde dehydrogenase; 11. Propionyl-CoA carboxylase; 12. Methylmalonyl-CoA mutase. Abbreviations: FAD+  = flavin adenine dinucleotide; GDP = guanosine diphosphate; GTP = guanosine triphosphate; NAD+  = nicotinamide adenine dinucleotide; TCA = tricarboxylic acid

The synthesis of valine occurs in microorganisms and plants, but not in animals [5]. Valine biosynthetic pathway consists of four steps (Fig. 1B). Pyruvate is the starting point of valine biosynthetic pathway, which arises from the glycolytic pathway. In the first step, pyruvate is converted to α-acetolactate by acetohydroxy acid synthase. In the second step, acetohydroxy acid isomeroreductase catalyzes the conversion of α-acetolactate to α,β-dihydroxyisovalerate, via auto-displacement of methyl groups. Dihydroxy-acid dehydratase is the enzyme for the third step in which α-ketoisovalerate is formed from α,β-dihydroxyisovalerate. It is worth noting that this enzyme is inhibited by valine [28]. In the last step, α-ketoisovalerate is catalyzed by branched-chain amino acid transaminase (BCAT) to synthesize valine. In industrial production, valine has been usually manufactured by the industrial mutant strains of *Corynebacterium glutamicum* and *Escherichia coli* (*E*. *coli*) [29]. Nowadays, some mutants or engineered strains may serve as specific probiotics for a production of valine or other amino acids to meet swine demand [30–33].

Unlike the synthetic pathway, valine could be catabolized through a similar process in all life-forms (Fig. 1C) [5]. Through a series of reactions, valine is eventually converted into succinyl-CoA, which enters the tricarboxylic acid cycle [34]. Moreover, it is mentionable that, as a precursor for the synthesis of branched chain fatty acids, valine is converted into *iso*-14:0 and *iso*-16:0, which are the main components of membrane lipids of gut bacteria [35, 36]. Likewise, leucine and isoleucine are also converted into *iso*-15:0, *iso*-17:0, and *anteiso*-15:0, *anteiso*-17:0, respectively [36].

### Interaction of valine with amino acids

#### Valine with isoleucine and leucine

**Fig. 2:**
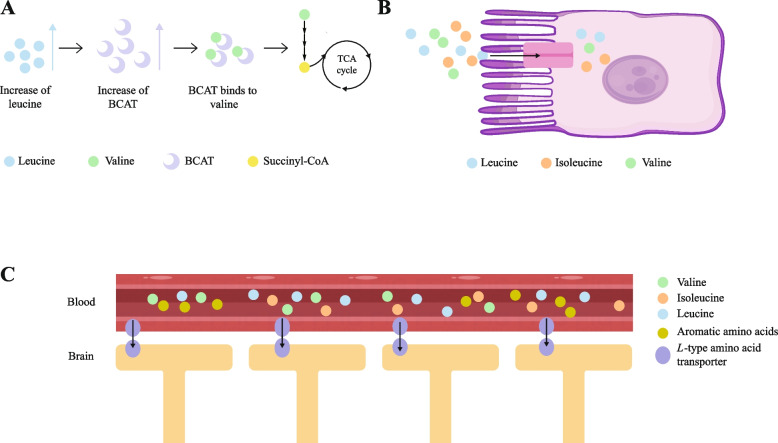
Interaction of valine with amino acids. **A** Valine competes with leucine for branched-chain amino acid transaminase (BCAT). High leucine competes for the BCAT and enhance the catabolism of valine. **B** Valine competes with leucine and isoleucine for the amino acid transporters in intestinal epithelial. **C** The BCAAs (leucine, isoleucine and valine) and aromatic amino acids, compete for transport into the brain through *L*-type amino acid transporters

Valine, leucine, and isoleucine belong to BCAAs and use common transport systems for amino acid absorption due to the similar structure of their side chains [5, 37]. In the metabolic pathway, BCAAs share enzymes for catabolizing the first two steps, the BCAT and branched chain keto-acid dehydrogenase [38]. Because the corn and corn by-products, such as distillers dried grains with solubles, have relatively high leucine concentrations, it is often possible that leucine is in excess in corn-based diets and practical high protein diets [39–42]. High leucine could stimulate the activity of metabolic enzymes and enhance the catabolism of valine thus reduce the serum concentration of valine (Fig. 2A) [43]. Likewise, because of the common transport systems, leucine and isoleucine could compete for the amino acid transporters and inhibit the absorption of valine [44] (Fig. 2B).

The antagonistic interactions among BCAAs in dietary have been observed in some species, like chicks, turkey poults, pigs, kittens, rats, and humans [45–48]. For six-week-old female pigs fed leucine (65% greater than the NRC 1998 requirement estimate), compared to supplementation with 0.18% valine, the effect of feeding the deficient valine diet (0) on plasma concentration of valine and feed intake after ingestion were examined [49]. The results showed that the deficient valine diet resulted in a 14% reduction in feed intake occurred within 1 h and reduced plasma concentration of valine, which might indicate the BCAA unbalance or deficiency [50]. In neonatal piglets, Elango et al. [51] reported that the BCAA antagonism could be relieved when the ratio was 1.2:1.8:1 (valine/leucine/isoleucine) in diet, and the mean total requirement of BCAA in parenteral was 56% of in enteral through breakpoint analysis.

On the one hand, in the high leucine diet of piglets, adding extra valine could largely counteract the feed intake reduction and growth performance decline caused by excess leucine, most likely because the valine addition diminishes the leucine uptake through the blood–brain barrier [52–54]. Nevertheless, valine supply could not correct the negative effect of excess leucine on the expression of b^0,+^, which is the most important transporter for cationic amino acid expressed in epithelial cells [55]. On the other hand, for carcass traits and meat quality in finishing pigs, valine and isoleucine had significant interactions in backfat thickness, water distribution forms and myofibrillar protein solubility [56]. Richert et al. [57] did not observe look at the interactions between isoleucine and valine in sows. Therefore, in terms of nutritional requirements, more experiments are needed to demonstrate the interaction between valine and isoleucine. Interestingly, valine oversupply is less unlikely to induce the BCAA antagonism compared with leucine and isoleucine, probably because valine is less important in BCAA antagonism, and excessive valine seem to have a lesser effect on increasing the catabolism of the other BCAA [9, 14, 58]. Burnham et al. [59] found that excess leucine reduced food intake in broilers, while excess valine had no effects.

#### Valine with aromatic amino acids

BCAAs and aromatic amino acids are large neutral amino acids, which share the *L*-type amino acid transporters, to compete for pass the blood–brain barrier (Fig. 2C) [60, 61]. When a lot of aromatic amino acids pass through the blood–brain barrier, the concentrations of various amines are increased, like tyramine and serotonin, which impairs brain function [62, 63]. BCAAs supplementation in diet may protect barrier function by inhibiting the passage of aromatic amino acids across the blood–brain barrier [64–66]. In the diet of older growing pigs, high levels of neutral amino acids enhanced tryptophan deficiency, resulting in decreased feed intake and growth performance [67]. Supplementation of sufficient valine (0.1%) in marginal tryptophan diet (0.004%) enhanced the body weight, while supplementation of sufficient tryptophan (0.054%) in marginal valine diet (0) decreased the body weight. In addition, the pigs in the low CP group (17.8%) showed the same performance as the high CP group when sufficient valine (0.1%) and tryptophan (0.054%) were added. It was confirmed that dietary valine and tryptophan levels significantly interact with each other on body weight gain of piglets [24].

### Biological functions of valine in swine

**Fig. 3:**
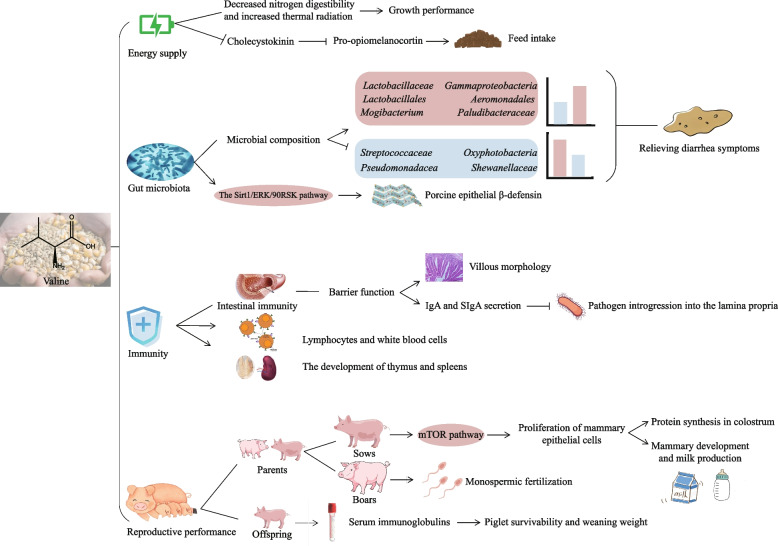
The roles of valine in swine nutrition and whole-body homeostasis. Apart from serving as an energy source, valine has multiple healthy functions, like improving the structure of gut microbiota, immune functions and reproductive performances. Abbreviations: Ig = immunoglobulin; mTOR = mammalian target of rapamycin; Sirt1/ERK/90RSK = sirtuin 1/the extracellular signal-regulated kinase/p90 ribosomal S6 kinase

As essential amino acids in livestock, BCAAs show special nutritional effects [10]. Valine plays positive roles in swine to regulate energy supply, gut microbiota structure, immune functions, and reproductive performance, whose biological functions have been reviewed in this part (Fig. 3).

#### Energy supply

Valine is one of the most efficient energy-generating amino acids through the oxidation of branched-chain α-keto acid dehydrogenase complex [68]. As the primary end product of valine metabolism, β-hydroxyisobutyrate is an ideal gluconeogenic substrate and a key indicator for the fate of valine in the muscle [38]. For mammals, valine also participated in glutamine synthesis [69]. Under starvation conditions, transaminase activities and amino acid oxidation rate are significantly enhanced, resulting in accelerated oxidation of BCAAs [70, 71].

In addition, it has been shown in fishes and mice that valine could influence appetite by regulating the expression of neuropeptides or hormones in the hypothalamus [72, 73]. Similarly, the important links between valine deficiency and appetite regulation were demonstrated in pigs [74]. In pig farms, valine deficiency could cause the suppressive effect on feed intake due to an excess supply of leucine, which is through decreasing the absorption of large neutral amino acid and overstimulating the mTOR signaling pathway [14, 43]. As a prominent signal to interfere with the regulation of neurons in the hypothalamic, cholecystokinin may be transported to the blood to activate pro-opiomelanocortin neuron involved in feeding behaviors [75–78]. Evidence shows that high valine content could reduce the cholecystokinin expression in the gastric fundus. After feeding at the standardized ileal digestible (SID) valine:lysine ratio of 0.65 in a reduced protein diet, compared with the SID valine:lysine ratio of 0.45, cholecystokinin expression was significantly down-regulated, resulting in the lower expression of pro-opiomelanocortin and the improved feed intake, which increased growth performance in weaned piglets [79].

Under severe protein restriction (CP ≤ 14%), supplemental valine improves the growth performance in pigs [80, 81]. A combination of isoleucine at NRC level and added valine above NRC level in weaned piglet diet for 35 d increased thermal radiation and decreased the digestibility of nitrogen, as well as recovered the inhibitory effects of very-low-protein diet on feed intake and growth performance [25, 82]. Optimum dietary valine could also improve growth performance by regulating lipid metabolism [83]. In IPEC-J2 cells, supplemental valine could stimulate triglyceride synthesis by increasing 3-hydroxyisobutyrate concentration, which is the only valine metabolites that could survive mitochondrial oxidation, and may promote fatty acid transport via upregulation of the fatty acid transporter mechanism [84].

#### Regulation of gut microbiota

Recently, mounting evidences have unveiled that gut microbiota plays a crucial role in BCAAs metabolism, including valine [85]. Several studies have investigated the correlation between gut microbiota composition and dietary valine level in livestock [81, 82, 86].

On the one hand, as a part of the intestinal protective barrier, host defense peptides produced by the gut mucosa could defend against pathogens and clear inflammation [87, 88]. In the swine, addition BCAAs (valine:leucine:isoleucine = 0.2 mmol/L:0.1 mmol/L:0.8 mmol/L) improved the immune defense ability by stimulating the expression of porcine epithelial β-defensin in the Sirt1/ERK/90RSK signaling pathway. The result was similar in intestinal porcine epithelial cells (IPEC-J2) [89]. This suggests that valine could be used as a means of nutritional intervention to induce the production of endogenous host defense peptides in swine, to resist the invasion of pathogen, like *Salmonella* Typhimurium, *Listeria monocytogenes* and *Erysipelothrix rhusiopathiae* [90].

On the other hand, valine may be involved in regulating gut microbial species [91]. Apart from the antibiotic therapy, one strategy to ameliorate post-weaning diarrhea is to alter the dietary protein quantity and quality to manipulate gastrointestinal structure and function [92]. Excessive proteins always induce diarrhea in piglets, with the increased relative abundances of *Fusobacterium* and Proteobacteria and some potentially toxic substances, including ammonia and indoles in the intestine [35, 93–96]. In protein restricted piglets, the proliferation of *Gammaproteobacteria*, *Lactobacillales*, and *Aeromonadales* was observed by supplementing valine (0.80%) and leucine (1.43%) for 35 d, as well as markedly enhanced the feed intake and the body weight gain [81]. Another evidence supported that the improvement of valine (0.44%) in LP diet on growth performance was associated with the high population of *Mogibacterium* in colon content [82]. The nonmetric multidimensional scaling showed the significant separation and cluster between control group and LP group supplementing with BCAAs (valine:leucine:isoleucine = 0.57:0.82:0.55) in nursery pigs. The supplemental BCAAs diet regulated the fecal microbiota composition, by increasing the abundances of *Paludibacteraceae* and *Synergistaceae* and reducing the abundances of *Streptococcaceae*, *Oxyphotobacteria_*unclassifed, *Pseudomonadaceae,* and *Shewanellaceae* [97].

Noteworthily, valine could be used as a chemical building block to enhance phagocytosis of macrophages against drug-resistant pathogens [98]. Antimicrobial peptide G6 is rich in valine and arginine residues, which decreases the bacterial load by 103-fold in sepsis mice and increases survival after 7 d of *Salmonella* Typhimurium infection [99, 100]. Similarly, exogenous valine had the capacity to activate the PI3K/Akt1 pathway and reduced the load of multidrug-resistant pathogens in mice [98].

#### Immunity

As the essential substrates of protein biosynthesis, BCAAs could promote intestinal development and enterocyte proliferation [101]. The absence of BCAAs impairs the innate immune function by decreasing lymphocytes and white blood cells [102–104]. Besides, the deficiency of BCAAs caused atrophy of the thymus and spleens [105, 106]. In the BCAAs, valine resulted in impairment of bone metabolism in particular [107]. The valine deficiency reduces the lymphocyte proliferation and impedes the growth of thymus and peripheral lymphoid tissue [108–110].

Secretory immunoglobulin A (SIgA) is an immunoglobulin (Ig) in the intestinal lumen. The secretion of SIgA was stimulated by BCAAs to thereby inhibiting pathogens into the lamina propria [103, 104]. In a protein restricted diet (17% CP) of weaned piglets, supplemented with BCAAs (valine:isoleucine:leucine = 0.27%:0.19%:0.07%) improved intestinal immune defense function via protecting villous morphology and increasing IgA levels in jejunum and ileum [111]. Similarly, valine participates in immune functions by increasing the concentration of IgM in serum of sows [112]. A study used CD14 positive monocytes isolated from peripheral blood mononuclear cells and supplemented valine for hepatitis C virus cirrhotic patients, which increased cytokine production and the allostimulatory capacity of human monocyte-derived dendritic cells [113].

Nutrition and immunoglobulins are obtained by newborn piglets from ingested colostrum, which are used for the development of the systemic immunity in piglets [114, 115]. Studies have shown that within 24 h of sow delivery, compared with colostrum-fed group, colostrum-deprived group still decreased concentrations of IgG in plasma and IgA in feces even though they normally suckle milk after 24 h [116]. As the continuation of the mother–infant bond, colostrum protects the piglets until their own immune system sufficiently matured to respond to foreign antigens [117]. Improved immune function and nutrition-related physiological function were observed in sows fed diet at valine:lysine ratio of 0.87. In their offspring, serum albumin concentration was also increased [112]. Moreover, sows are more likely to be active in the loose-housed and free-farrowing system, resulting in low survival rate of piglets by crushing [118]. Evidence in the loose housing suggested that the heavy piglets had a higher concentration of valine in serum than the lighter littermates [119].

#### Reproductive performance

Sow milk provides a large range of nutrients, bioactive compounds and beneficial microorganisms [120–123]. Sow nutrition plays an important role in mammary development and milk production of sows, which affects the survivability and weaning weight of piglets [124, 125]. Among all amino acids, the uptake of BCAAs exceeds the output in milk in the mammary gland of lactation sows. In lactating porcine mammary tissue, BCAAs are mainly further metabolized into glutamine and aspartate that are abundant amino acids in milk protein [69, 126]. Compared with stage of gestation, the perinatal period of energy requirements increases by 60%, and as well as amino acid requirements more than double in sows [127, 128]. Notably, at the tissue level, other amino acids such as leucine inhibit the uptake of valine in sow mammary tissue [129].

The high absorption of valine during lactation suggested that valine may play an important role in the metabolism of mammary gland [130]. For sows, the total dietary valine:lysine ratio at 0.99:1 also enhanced valine concentration in milk [131]. Valine effectively regulated mobilization of body reserves for lactation sows with high feed intake. With the number of weaned piglets elevating, the valine requirements in milk and mammary gland tissue also increase [22, 132]. It was confirmed that the addition of 0.116 mmol/L valine promoted the monospermic fertilization and stimulated male pronuclear formation after maturation [133]. Moreover, the exposure of porcine mammary epithelial cell to 0.9 mmol/L valine increased the proliferation of porcine mammary epithelial cells, and promoted protein synthesis in colostrum via the mammalian target of rapamycin signaling pathway [134]. Growth of neonate is partly dependent on the sufficient protein content of sow milk [135]. During late gestation in gilts, compared with the first day of lactation, dietary valine addition from 63% to 93% linearly increased the protein synthesis and the fat synthesis in colostrum on the tenth day, which increased from 0.01% to 26.3% and from 1.3% to 72.4%, respectively [136]. Elevating valine level from 0.8% to 1.2% in sow diet led to higher weaning weight of piglets [137]. The valine concentration requirement of lactation sows is supposed to exceed 6.5 g/kg to avoid seriously decreasing feed intake and milk yield of sows and growth performance of piglets [138].

Since lactation is a period of high metabolic load, sows are sensitive to ambient temperature [139]. Heat stress commonly induces oxidative stress and protein metabolism imbalance [140–142]. A good deal of conducted research indicated substantial quantities of fertility and reproductive problems within the heat-stressed sows [143]. Heat stress is usually along with the long intervals from weaning to estrus, low farrowing rates, depressed litter size, and reduced milk production, which eventually had negative effects on piglet growth and weaning weight [144–146]. Under heat stress, valine is a potential agent for alleviating seasonal infertility of sows by improving feed intake and increasing the concentration of lactose in colostrum [112].

### Valine requirements in different stages of swine

In pig diet, lysine, threonine, methionine, and tryptophan have been acknowledged as limiting amino acids [21]. Recently, valine is considered to be the next limiting amino acid in swine [21, 147]. It has to be kept in mind that the amino acid requirements vary with the duration of diet regimen, genetic background, and physiological status of pigs [98, 148]. The nutrient requirements of valine in different experiments were summarized in Table 2 [24, 79, 80, 82, 138, 149–159]. The recommended valine requirement for weaned piglets is 64% in the NRC 2012 [25]. For the same CP level at 17.7% in weaned piglets, Wiltafsky et al. [151] assumed that the SID valine:lysine ratio of 66% could achieve optimal average daily gain (ADG), while Jansman et al. [24] reported that when the SID of valine:lysine increased from 67% to 75%, the better responses of feed intake and body weight gain were achieved. Additionally, the predicted valine requirements in piglets are also varied in different computational models. In a quadratic polynomial model, Clark et al. [160] estimated that when the dietary SID valine:lysine ratio was 71.7%, the ADG:average daily feed intake might reach the maximum. According to the response curves of weaned piglets in the LP diet (14.64% CP), the SID valine:lysine requirements were predicted as 67.7% and 71.7% for high feed conversion rate, in the linear-plateau and curvilinear-plateau models, respectively [149]. In the future, based on existing standards and the accumulation of subsequent trail, researchers need to further optimize and refine the nutritional requirements of swine, so as to adapt to various physiological and environmental conditions, such as different genders, breeds, seasons, and regions.

**Table 2:** Comprehensive summary of the existing literature on the relationship between valine and performance in swine

Nutrition treatment	Category	Breed	Experiment period/ages	Effect	References
70% SID valine/lysine ratio	Post-weaned piglets	Pietrain × (Large White × Landrace)	43-day-old to 64-day-old	Improving ADFI and ADG	[24]
67%–75% SID valine/lysine ratio	Post-weaning piglets	(Great Yorkshire × Pietrain) × Dalland	34-day-old to 72-day-old	Increasing body weight gain feed intake	[79]
68% SID valine/lysine ratio	Post-weaned piglets	(Duroc × Landrace) × Pietrain	31-day-old to 73-day-old	Improving the piglet weight gain and feed intake	[80]
66% SID valine/lysine ratio	Weaned piglets	Crossbred piglets	31-day-old to 65-day-old	Getting optimal ADG	[82]
65% SID valine/lysine ratio	Weaned piglets	Duroc × Landrace × Large White	14-day experimental period	Increasing ADFI, ADG and FCR; decreasing plasma urea nitrogen, CCK and POMC expression	[138]
0.31% dietary valine	Weaned piglets	Duroc sire line and Large White × Landrace dam	35-day experimental period	Restoring the reduced transcript of IGF-1; improving gut morphology	[149]
0.44% dietary valine	Weaned piglets	Duroc sire line and Large White × Landrace dam	21-day-old to 56-day-old	Improving ADFI; increasing the abundance of *Mogibacterium* in colon	[150]
69% SID valine/lysine ratio	Growing pigs	No data	No data	Increasing ADG by 5%	[151]
11.25 g/d valine	Finishing pigs	Approximately 75% Large White × Landrace and 25% Duroc × Hampshire	14-day experimental period	Increasing weight gain and feed efficiency	[152]
88% SID valine/lysine ratio	Lactating sows	Large White × Landrace	From d 107 of gestation to d 28 of lactation	Increasing the concentrations of all the amino acids in sow colostrum; having a tendency to increase IgM in sow colostrum; increasing ADG of piglets	[153]
Dietary valine concentration of 6.5 g/kg	Lactation sows	German Landrace	From d 84 of gestation to d 35 of lactation	Increasing feed intake and milk yield of sows and growth performance of piglets	[154]
0.68% dietary valine	Lactating sows	Landrace × Yorkshire	A 21-day lactation	Maximizing total solids production and protein production	[155]
0.85% dietary valine	Lactation sows (particularly nursing large litters)	German Landrace	Farrowing to d 35 of lactation	Increasing amino acid concentrations in milk; decreasing urea concentrations in blood plasma	[156]
Valine: lysine ratio of 100%	Sows weaned fewer than 10 pigs	Large White × Landrace × Yorkshire	No data	Increasing litter weight gain	[157]
Above 1.15% of the diet (72 g/d of valine intake)	High-producing sows (21-day litter weights > 60 kg)	Large White × Landrace or Large White × Chester White × Landrace	Approximately d 110 of gestation to d 26 of weaning	Increasing litter weaning weight and litter weight gain	[158]
Total valine:lysine = 0.93:1 (18.33 g/d from d 75 to 90 of gestation; 21.49 g/d from d 91 to farrowing)	Gilts	Yorkshire × Landrace	From d 75 of gestation to d 21 of lactation	Increasing the concentration of milk fat and proportion of total saturated and monounsaturated fatty acids	[159]

*ADG* Average daily gain, *ADFI* Average daily feed intake, *CCK* Cholecystokinin, *FCR* Feed conversion ratio, *Ig* Immunoglobulin, *IGF* Insulin-like growth factor, *POMC* Pro-opiomelanocortin

Likewise, in LP diet, the growing-finishing pigs have the higher requirement for valine compared with traditional diets [21]. A study has shown that supplemented with 0.15% valine in a low CP diet (17% CP) had less severe diarrhea symptoms and contributed to the similar performance of ADG, as in a high CP diet (20% CP) in piglets [161]. However, a potential disadvantage of blindly reducing CP level is the decreased growth performance following weaning caused by a dietary imbalance of amino acid [20]. Much work remains to be done on the optimal nutrient requirements in the LP diet which valine would be most effective in improving growth performance in swine. Targeted application of valine to support animal demands should be valued to balance feed costs and breeding benefits.

The contents of the other two BCAAs also affect the valine requirements of pigs due to the antagonistic interactions among BCAAs. The high level of leucine potentially reduces serum valine concentration, which means that the high leucine diet tends to require more valine supplementation [43]. The addition of valine (7.8 g/kg) partially mitigated the decrease in feed intake caused by the high-leucine diet (21.3 g/kg), and greatly increased the daily gain and feed conversion rate of piglets [52]. Due to the plasma membrane transport system L, which is in many cells the only (efficient) pathway for BCAA, isoleucine competes for amino acid transporters, thereby inhibiting the absorption of valine [44, 61]. For carcass traits and meat quality in finishing pigs, high dietary valine intake undermined water holding capacity, decreased sarcoplasmic protein solubility and pH_24h_ value, whereas high dietary isoleucine increased pH_24h_ value, sarcomere length, suggesting that valine and isoleucine had significant interactions in backfat thickness, water distribution forms and myofibrillar protein solubility [56]. However, in terms of nutrient requirements, there are currently no experiments demonstrating the interaction between valine and isoleucine.

## Conclusions

Herein, metabolic pathways of valine and its interactions with other amino acids were summarized. We emphasized the biological functions and the nutrient requirements of valine at different growth stages in swine. Given that amino acid requirements vary with the duration of the diet regimen, genetic background and physiological state of pigs, the targeted application of valine is essential [98, 148]. In animal husbandry, supplementation with valine not only increases growth and reproductive performances, but also regulates gut microbiota and immune functions. Moreover, in the human brain, the deficiency of valine caused neurological defects and mental retardation [162]. Likewise, BCAA treatment induced reorganization of actin and cytoskeleton, particularly valine [163]. High concentration of BCAAs reduced the migration and invasion ability of breast cancer cells, which had a positive effect on the treatment of breast cancer [164]. In recent years, there has been a growing global demand for valine in animal feed, commercial medical treatment and industrial applications [165]. It would be worthwhile to further investigate their potential functionality in life science.

In pig production, some mutants or engineered strains were obtained for an overproduction of specific amino acids to meet the swine demand [31–33]. The efficient application of the valine has been advanced by engineered *Corynebacterium glutamicum* [166], *E*. *coli* [167], *Bacillus subtilis* [168], *Bacillus licheniformis* [169], *Saccharomyces cerevisiae* [165]. In animal nutrition, the appropriate *L*-valine produced by fermentation using *E*. *coli* CCTCC M2020321 or *E*. *coli* KCCM 80159 was added to the diet, which was safe for consumers, users and the environment, and the valine produced by fermentation also was used as an effective source of nutritionally essential amino acid *L*-valine in non-ruminant animals [170, 171]. However, considering that competitive pathways have the interdependency and metabolic burdens in engineered strains, it is a challenge to optimize high valine production for a single bacterial strain [172]. In the coming period, high-throughput biosensor screening would be instrumental in high-yielding valine producer strains [32, 172].

## Data Availability

Not applicable.

## Abbreviations

ADG: Average daily gain
BCAAs: Branched-chain amino acids
BCAT: Branched-chain amino acid transaminase
CP: Crude protein
*E. coli*: *Escherichia coli*
Ig: Immunoglobulin
LP: Low-protein
SID: Standardized ileal digestible
SIgA: Secretory immunoglobulin A
